# High anti-human cytomegalovirus antibody levels are associated with the progression of essential hypertension and target organ damage in Han Chinese population

**DOI:** 10.1371/journal.pone.0181440

**Published:** 2017-08-24

**Authors:** Zhen Li, Yan Tang, Na Tang, Qian Feng, Hua Zhong, Yong-min Liu, La-mei Wang, Fang He

**Affiliations:** 1 Department of Pathophysiology/Key Laboratory of Education Ministry of Xinjiang Endemic and Ethnic Diseases, Medical College of Shihezi University, Shihezi, China; 2 Department of Emergency and critical care medicine, the First Affiliated Hospital of Medical College of Shihezi University, Shihezi, China; 3 Department of Geriatrics, the First Affiliated Hospital of Medical College of Shihezi University, Shihezi, China; 4 Centre of Medical Functional Experiments, Medical College of Shihezi University, Shihezi, China; University of St Andrews, UNITED KINGDOM

## Abstract

Human cytomegalovirus (CMV) infection is associated with hypertension and has been linked with the pathogenesis of increased arterial blood pressure (BP). Currently, whether CMV infection is associated with the progression of hypertension and hypertensive target organ damage (TOD) remains to be identified. We aimed to examine the relationship between CMV infection and the progression of hypertension and hypertensive TOD, which could provide clues on the possible mediating mechanisms, in the Han Chinese population. A total of 372 patients with hypertension and 191 healthy controls (Han participants from Xinjiang, China) were included in the study. Enzyme-linked immunosorbent assay (ELISA) and qPCR were used to detect CMV infection. C-reactive protein (CRP), tumor necrosis factor-α (TNF-α), and interleukin-6 (IL-6) titers were also analyzed using an ELISA kit. Moreover, cardiovascular disease markers were evaluated by echocardiography, carotid ultrasonography, and tomographic scans. Essential hypertension (EH) patients exhibited a marked increase in CMV IgG antibody, CRP, TNF-α, and IL-6 levels. Higher grade of hypertension and hypertensive TOD had higher CMV IgG antibody and CRP levels. The CMV IgG antibody titers were positively correlated with arterial BP, greater grade of hypertension and hypertensive TOD, and CRP and IL-6 levels. The higher quartile of CMV IgG titer and CRP level were associated with the incidence of hypertension and the progression of hypertension and hypertensive TOD. In the Han Chinese population, high CMV IgG titers are associated with the progression of hypertension and hypertensive TOD. CMV IgG titer >4.25 U could be an independent predictor of hypertension and progression of hypertension, while that >4.85 U could be an independent risk factor for hypertensive TOD. The underlying mechanism may be largely mediated by chronic inflammation.

## Introduction

Hypertension is a global disease [1]. More than 1 billion adults have hypertension worldwide, and 90–95% of which is essential hypertension (EH) [2]. Several risk factors and features are involved in the development of hypertension, including age, race, gender, family history of hypertension (FH), environmental interactions, dietary factors, and stress [1, 3–6]. Although the definite cause of hypertension is poorly understood, preventive measures may contribute to decreasing blood pressure (BP) [7–9].

Human cytomegalovirus (CMV) infection plays an essential role in hypertension. CMV, a member of the beta-herpesvirus family, contains a large double-stranded DNA genome and is widespread [10]. CMV seroprevalence is 40% during people’s first year of life [11] and ranges from 40 to 100% in adults [12]. CMV seropositive rate is 72.3 and 65.9% in women and men, respectively, in Finland [13]; 66.7% in the USA [14]; and 90.1% in Hans and 95.4% in Kazakhs in Xinjiang Province, China [15]. Primary CMV infection has lifelong persistence, mostly an asymptomatic infection in a human host, and results in the development of a lifelong carrier status with periodic reactivation and shedding of the virus from mucosal sites [11, 16]. Previous studies demonstrated that CMV is associated with various diseases, including cardiovascular disease (CVD) [14, 15, 17–19], those affecting cognitive functioning [20, 21], tumor [22], and type 2 diabetes mellitus [3, 23], and even mortality [4, 24].

Moreover, CMV may mediate inflammatory responses. CMV IgG antibody up-regulation may increase inflammatory cytokines [4]. Elevated levels of C-reactive protein (CRP), tumor necrosis factor-α (TNF-α), and interleukin-6 (IL-6) are found to be associated with cytomegalovirus-related diseases [4, 14, 18, 25].

This study aimed to assess the relationship between CMV infection and the progression of hypertension and hypertensive target organ damage (TOD) among the Han Chinese population. We hypothesized that higher CMV IgG antibody levels is associated with the progression of hypertension and hypertensive TOD and that CMV infection and inflammatory factors are correlated.

## Material and methods

### Study participants and data collection

Here, 563 participants from the Shihezi and Shawan regions in Xinjiang Province, China, were recruited between November 2014 and July 2015. The study protocol was reviewed and approved by the Ethics Committee of First Affiliated Hospital of Medical College of Shihezi University (approval number: AF/SC-08/01.0) and performed in strict accordance with the rules ethics review of Biomedical research in human (2007) and the principles of the Declaration of Helsinki. All participants signed the informed consent, completed a questionnaire, accepted essential auxiliary examination, and agreed to have their blood samples collected for future research.

A total of 191 normotensives and 372 hypertensives were involved in the study. The participants were seated in a relaxed position for at least 15 min prior to BP measurement, which was performed three times. We used the 1999 WHO/ISH Hypertension Guidelines for the definition of hypertension: systolic BP (SBP) ≥140 mmHg and/or diastolic BP (DBP) ≥90 mmHg or use of antihypertensive agents. Hypertension was further divided into three grades (grade 1 = 140/90–159/99 mmHg, grade 2 = 160/100–179/109 mmHg, and grade 3 ≥180/110 mmHg). The exclusion criteria were (i) secondary hypertension; (ii) acute infection or illness in the last 4 weeks; (iii) organ transplantation, tumor, and immunodeficiency disease (including HIV infection) or autoimmune disease.

Obtaining medical history, physical examination, laboratory investigation, and further diagnostic tests were performed in all participants. Data on age, gender, and cardiovascular risk factors, such as smoking status, drinking status, FH, body mass index (BMI), fasting blood sugar (FBS), total cholesterol (TC), triglycerides (TG), low-density lipoprotein cholesterol (LDL-c), high-density lipoprotein cholesterol (HDL-c), CRP, TNF-α, and IL-6, were obtained. Blood samples were assessed for CMV infection, including CMV IgG and CMV IgM antibody and CMV DNA. Serum blood urea nitrogen, serum creatinine (Cr), inter-ventricular septum end-diastolic thickness (IVSd) and left ventricular end-diastolic dimension (LVIDd), carotid atherosclerotic plaques (CAP), white matter lesions, infarctions, and microbleeds were identified as CVD markers.

Hypertensive TOD was defined as a cardiovascular event that affects the following: (i) heart: myocardial infarction (at least two of the following diagnostic criteria: typical chest pain, ECG diagnosis, transient conventional myocardial enzyme increase by more than twice the normal), ventricular hypertrophy, and ischemic heart disease; (ii) arteries: carotid plaques; (iii) brain: stroke (cognitive decline, computer tomography evidence); and (iv) kidney: chronic kidney disease (CKD) [5, 26, 27].

### Enzyme-linked immunosorbent assay

Enzyme-linked immunosorbent assay kit (CMV IgG antibody Diagnostic Kit or CMV IgG antibody Diagnostic Kit, Haitai Biotech Inc, Zhuhai, China) was employed to determine CMV infection (IgG antibody, IgM antibody) using baseline frozen (-80°C) serum samples according to the product specifications (antibody titer <1.1 is seronegative; antibody titer ≥1.1, seropositive). The test sensitivity was 96.2 and 99.9% and the specificity was 97.8 and 99.8% for CMV IgG and IgM, respectively. The optical density (OD) value and the antibody titer were proportional. Among the 563 participants, 16 participants were CMV IgG seronegative (antibody titer <1.1) and 547 were CMV IgG seropositive (≥1.1); 561 participants were CMV IgM seronegative (<1.1) and 2 were CMV IgM seropositive (≥1.1). All participants were further categorized into four groups according to quartiles of CMV antibody concentrations (U): 0–3.75, 3.76–4.25, 4.26–4.85, and >4.85.

We also evaluated CRP, TNF-α, and IL-6 levels with E-EL-H0043c, E-EL-H0109c, and E-EL-H0102c, respectively (Wuhan Elabscience Biotechnology, Wuhan, China), according to the manufacturer’s instructions. The OD value and the CRP/TNF-α/IL-6 concentration were proportional. CRP/TNF-α/IL-6 concentrations in the samples were calculated based on the OD value and standard curve, according to the kit manual, using the software curve expert 1.3.

### DNA extraction and qPCR detection

Blood specimens were stored at -80°C until DNA extraction, which was performed using QIAGEN kit (QIAGEN Inc., Valencia, CA) according to the manufacturer’s directions. We evaluated the seropositivity and copy number of HCMV using CMV-DNA kit (DaAn Co, Guangzhou, China) in all participants; the PCR primers for this assay were derived from the immediate early gene. The PCR conditions were as follows: 93°C for 2 min, followed by 10 cycles of 93°C for 45 s, 55°C for 1 min, and 30 cycles of 93°C for 30 s and 33°C for 45 s. 7300 Real-Time PCR system (Applied Biosystems, Singapore) was employed. Real-time fluorescence measurements were recorded and the threshold cycle (Ct) value calculated by determining the point at which the fluorescence exceeded a threshold limit according to the manufacturer’s directions. Samples were considered positive when the Ct values exceeded 30 cycles.

### Doppler echocardiography

Doppler echocardiogram was performed using an EPIQ 7C B-mode ultrasonography (EPIQ 7C, Philips Ultrasound Inc., USA). IVSd and LVIDd were identified based on the American Society of Echocardiography recommendations. Patients were examined in a resting position for specific parts of the examination and during end-expiration by a single experienced examiner with no access to the clinical data of the participants.

### Carotid ultrasonography

The common carotid artery was examined by ultrasonography (EPIQ 7C, Philips Ultrasound Inc., USA). The subject was placed in the supine position with his/her neck extended and rolled contralaterally by approximately 45°. The right/left common carotid artery was examined at 1.5 cm proximal to the carotid bifurcation. Subjects with a focal encroachment of the carotid arterial wall and area of focal protrusion into the lumen at least 50% greater than the surrounding wall thickness were diagnosed as having carotid plaque [28, 29]. All carotid ultrasound examinations were performed by the same assessor who had no access to the subjects’ clinical data.

### Computed tomography of the brain

All subjects were examined using computed tomography (CT) (GT 1700V, GE Healthcare, USA). To identify the predictors, CT scan was performed and the finding (dependent variable) was categorized as normal or abnormal. Abnormal CT scan includes the detection of white matter lesions, silent brain infarctions, lacunar infarctions, or microbleeds. Two assessors performed the evaluation, who were blinded to the clinical findings.

### Statistical analysis

For continuous variables, the t test and Kruskal-Wallis ANOVA (for normally distributed variables) or Mann-Whitney U test (for skewed variables) was employed. For categorical variables, the chi-squared test was used. Multivariable linear regression was performed to assess the association between the CMV IgG titers or inflammatory cytokines and BP values, and was further used to identify the relationship between inflammatory cytokine levels and CMV IgG titer values. Spearman’s rank correlation and partial correlation analyses were performed to evaluate the association between CMV IgG titers and the different grades of BP or hypertension without/with TOD and that between inflammatory cytokines and the different grades of BP or hypertension without/with TOD. Subsequently, we evaluated the relationship between CMV and risk of hypertension and hypertensive TOD (as a dependent variable) using binary logistic regression. Moreover, we analyzed the association of CMV with the risk of hypertension according to the grade by ordinal logistic regression. *P*<0.05 was considered statistically significant. All statistical analyses were performed using SPSS version 20.0 (IBM Corporation, Armonk, NY).

## Results

A total of 563 subjects (28–86 years old) were randomly selected. Table 1 lists the participants’ clinical characteristics stratified by BP. Participants were further categorized into two (normotensive and hypertensive) or four (normotension and hypertension grades 1 to 3) groups according to the BP values. The number of normotensive subjects and those with grade 1, grade 2 and grade 3 hypertension was 191, 110, 114 and 148, respectively. The participants with hypertension were older (*P*<0.001); were more likely to be males (*P* = 0.006); had higher weight, BP (including SBP, DBP, and mean arterial pressure(MAP)), and CMV IgG antibody, CRP, TNF-α, and IL-6 levels (CRP, *P* = 0.005; IL-6, *P* = 0.025; others, *P*<0.001); had higher levels of other traditional CVD risk factors, such as BMI, FBS, and TG (*P*<0.001); and had lower levels of HDL-C (*P* = 0.021). BUN, Cr, IVSd, and LVIDd values and the incidence of brain infarction and CAP were also higher (BUN, *P* = 0.020; brain infarction incidence, *P* = 0.006; others, *P*<0.001) in the hypertension group than in the control group.

**Table 1:** Clinical characteristics and laboratory values of study participants by BP categories.

Characteritics	Normotensive	Hypertension	*P*_*1*_ value	Grade 1 hypertension	Grade 2 hypertension	Grade 3 hypertension	*P*_*2*_ value
**n**	191	372		110	114	148	
**Age (years)**	48.77±8.68	58.79±12.18	<0.001*	54.97±11.11*	60.31±12.01*^#^	60.45±12.50*^#^	<0.001
**Males (%)**	66 (34.55)	174 (46.77)	0.006*	59 (53.64)*	50 (43.86)	65 (43.92)	0.013
**Smokers (%)**	67 (35.08)	130 (34.95)	0.975	47 (42.73)	37 (32.46)	46 (31.08)	0.240
**Alcohol (%)**	46 (24.08)	93 (25.00)	0.811	33 (30.00)	29 (25.44)	31 (20.95)	0.414
**FH (%)**	41 (21.47)	99 (26.61)	0.181	38 (34.55)	25 (21.93)	36 (24.32)	0.064
**Weight (Kg)**	67.63±11.58	73.43±11.94	<0.001*	73.63±12.08*	70.93±11.20	75.20±12.12*^&^t1ad	<0.001
**Height (m)**	1.66±0.08	1.67±0.08	0.588	1.68±0.08	1.65±0.08	1.67±0.09	0.207
**BMI (kg/m**^**2**^**)**	24.34±2.99	26.31±3.01	<0.001*	26.02±2.56*	25.82±2.94*	26.91±3.27*^&^	<0.001
**SBP (mmHg)**	120.66±7.41	165.76±17.77	<0.001*	144.67±8.52*	164.02±5.96*^#^	182.77±9.46*^#^^&^	<0.001
**DBP (mmHg)**	74.77±6.11	97.08±12.48	<0.001*	85.40±2.93*	96.24±7.94*^#^	106.41±12.15*^#^^&^	<0.001
**MAP (mmHg)**	90.06±6.07	119.93±12.63	<0.001*	105.17±2.96*	118.81±5.44*^#^	131.76±8.28*^#^^&^	<0.001
**CMV IgG antibody titers (U)**	3.56±1.07	4.69±1.49	<0.001*	4.02±0.87*	4.26±1.32*	5.51±1.59*^#^^&^	<0.001
**CMV IgG seropositivity (%)**	186 (97.38)	361 (97.04)	0.819	105 (95.45)	110 (96.49)	146 (98.65)	0.464
**CMV IgM antibody titers (U)**	0.25±0.20	0.24±0.12	0.656	0.24±0.13	0.24±0.12	0.24±0.10	0.972
**CMV IgM seropositivity (%)**	1 (0.52)	1 (0.27)	0.631	0 (0)	1 (0.88)	0 (0)	0.583
**CMV DNA-positive (%)**	1 (0.52)	4 (1.08)	0.507	0 (0)	1 (0.88)	3 (2.04)	0.322
**CRP (ug/ml)**	0.50 (0.20–0.91)	0.56 (0.22–1.23)	0.005*	0.53 (0.30–0.86)	0.53 (0.29–1.03)*^#^	0.64 (0.22–1.23)*^#^^&^	<0.001
**TNF-α (ng/ml)**	0.18 (0.13–0.53)	0.19 (0.13–0.48)	<0.001*	0.19 (0.13–0.45)*	0.20 (0.13–0.48)*	0.19 (0.13–0.36)*^#^	<0.001
**IL-6 (ng/ml)**	0.16 (0.05–0.31)	0.16 (0.05–0.49)	0.025*	0.16 (0.05–0.33)	0.14 (0.05–0.49)	0.17 (0.09–0.45)*^#^^&^	<0.001
**FBS (mmol/L)**	4.95±0.64	5.41±0.94	<0.001*	5.19±0.71	5.22±0.79*	5.71±1.11*^#^^&^	<0.001
**TC (mmol/L)**	4.38±1.00	4.24±0.96	0.103	4.21±0.91	4.25±0.97	4.25±0.99	0.433
**TG (mmol/L)**	1.17±0.53	1.67±0.85	<0.001*	1.53±0.57*	1.63±0.92*	1.81±0.96*^#^	<0.001
**LDL-C (mmol/L)**	2.82±0.78	2.72±0.87	0.182	2.69±0.76	2.72±0.85	2.74±0.95	0.567
**HDL-C (mmol/L)**	1.17±0.28	1.11±0.33	0.021*	1.12±0.30	1.14±0.41	1.07±0.27*	0.041
**BUN (mmol/L)**	5.12 (2.04–8.50)	5.31 (1.98–91.40)	0.020*	5.53 (2.60–9.97)*	5.30 (2.43–91.40)	5.15 (1.98–68.00)	0.020
**Cr (umol/L)**	65.10 (43.10–107.10)	70.40 (45.20–588.70)	<0.001*	72.50 (45.20–201.70)*	70.40 (45.20–588.70)*	69.30 (45.20–381.2)*	<0.001
**IVSd (mm)**	8.39±1.06	9.07±1.09	<0.001*	9.04±1.00*	8.78±1.08*	9.33±1.10*^&^	<0.001
**LVIDd (mm)**	8.46±1.02	9.30±1.13	<0.001*	8.97±1.01*	9.29±1.13*	9.55±1.14*^#^	<0.001
**Brain infarction (%)**	27 (14.14)	168 (45.16)	0.006*	43 (39.09)*	47 (41.23)*	78 (52.70)*^#^	<0.001
**CAP (%)**	42 (21.99)	200 (53.76)	<0.001*	43 (39.09)*	63 (55.26)*^#^	94 (63.51)*^#^	<0.001

Values are given as mean ± SD, percentages or median [range]. Abbreviations: BP, blood pressure; FH, familial history of hypertension; BMI, body mass index; SBP, systolic blood pressure; DBP, diastolic blood pressure; MAP, mean arterial blood pressure; CMV, cytomegalovirus; CRP, C-reactive protein; TNF-α, tumor necrosis factor-α; IL-6, interleukin-6; FBS, fasting blood sugar; TC, total cholesterol; TG, triglyceride; LDL-C, low-density lipoprotein cholesterol; HDL-C, high-density lipoprotein cholesterol; BUN, serum blood urea nitrogen; Cr, serum creatinine; IVSd, the inter-ventricular septum end-diastolic thickness; LVIDd, the left ventricular end-diastolic dimension; CAP, carotid atherosclerotic plaques. *P*_*1*_ value, hypertension versus (vs.) normotensive; *P*_*2*_ value, grade 3 hypertension vs. grade 2 hypertension vs. grade 1 hypertension vs. normotensive, respectively.

*P<0.05, hypertension, grade 1, grade 2 or grade 3 hypertension vs. normotensive, respectively

^#^*P*<0.05, grade 2 or grade 3 hypertension vs. grade 1 hypertension, respectively

^&^*P*<0.05, grade 3 hypertension vs. grade 2 hypertension.

Furthermore we examined whether differences among the four categories of BP exist. Participants with a higher grade of BP had higher CRP levels (*P*<0.001) than those with lower grade of BP. Those with grade 2 or grade 3 hypertension were older (*P*<0.05) and had higher CAP value (*P*<0.05), while those with grade 3 hypertension had higher CMV IgG, IL-6 and FBS values (*P*<0.05) than those with grade 1 or grade 2 hypertension; had higher TG and LVIDd values (*P*<0.05) and higher incidence of brain infarction than those with grade 1 hypertension; and had higher weight, BMI, and IVSd value than (*P*<0.05) those with grade 2 hypertension. No significant differences in smoking, drinking, FH, TC, LDL-c, CMV IgM antibody titers, CMV IgG and IgM seroprevalence, and CMV DNA among the subjects (Table 1). At baseline, 86 participants had hypertension without TOD, and 286 had hypertension with TOD. The participants with hypertension with TOD were more likely to be older (*P*<0.05) and had higher BP (including SBP, DBP, and MAP), CMV IgG antibody, CRP, IVSd and LVIDd values (*P*<0.05) than those with hypertension without TOD (Table 2).

**Table 2:** Clinical characteristics and laboratory values of study participants in normotensive and hypertension with/without TOD.

Characteritics	Normotensive	Hypertension without TOD	Hypertension with TOD	*P* value
**n**	191	86	286	
**Age (years)**	48.77±8.68	51.31±8.34	61.03±12.26*^#^	<0.001
**Males (%)**	66 (34.55)	40 (46.51)*	134 (46.85)*	0.021
**Smokers (%)**	67 (35.08)	30 (34.88)	100 (34.97)	0.999
**Alcohol (%)**	46 (24.08)	28 (32.56)	65 (22.73)	0.065
**FH (%)**	41 (21.47)	28 (32.56)	71 (24.83)	0.174
**Weight (Kg)**	67.63±11.58	72.99±11.08*	73.56±12.20*	<0.001
**Height (m)**	1.66±0.08	1.68±0.08	1.66±0.08	0.522
**BMI (kg/m**^**2**^**)**	24.34±2.99	25.93±2.81*	26.43±3.06*	<0.001
**SBP (mmHg)**	120.66±7.41	156.37±18.17*	168.58±16.68*^#^	<0.001
**DBP (mmHg)**	74.77±6.11	93.90±10.70*	98.04±12.82*^#^	<0.001
**MAP (mmHg)**	90.06±6.07	114.72±12.07*	121.49±12.39*^#^	<0.001
**CMV IgG antibody titers (U)**	3.56±1.07	4.16±1.16*	4.85±1.54*^#^	<0.001
**CMV IgG seropositivity (%)**	186 (97.38)	82 (95.35)	279 (97.55)	0.545
**CMV IgM antibody titers (U)**	0.25±0.20	0.23±0.09	0.24±0.12	0.714
**CMV IgM seropositivity (%)**	1 (0.52)	0 (0)	1 (0.35)	0.795
**CMV DNA-positive (%)**	1 (0.52)	0 (0)	4 (1.40)	0.384
**CRP (ug/ml)**	0.50 (0.20–0.91)	0.53 (0.34–0.88)	0.58 (0.22–1.23)*^#^	0.003
**TNF-α (ng/ml)**	0.18 (0.13–0.53)	0.18 (0.13–0.45)*	0.20 (0.13–0.48)*	<0.001
**IL-6 (ng/ml)**	0.16 (0.05–0.31)	0.16 (0.05–0.29)	0.16 (0.05–0.49)*	0.035
**FBS (mmol/L)**	4.95±0.64	5.28±0.73*	5.45±0.99*	<0.001
**TC (mmol/L)**	4.38±1.00	4.23±0.93	4.24±0.97	0.264
**TG (mmol/L)**	1.17±0.53	1.51±0.54*	1.72±0.92*	<0.001
**LDL-C (mmol/L)**	2.82±0.78	2.60±0.81	2.76±0.88	0.119
**HDL-C (mmol/L)**	1.17±0.28	1.15±0.30	1.09±0.34*	0.025
**BUN (mmol/L)**	5.12 (2.04–8.50)	5.22 (2.60–8.20)	5.36 (1.98–91.40)	0.051
**Cr (umol/L)**	65.10 (43.10–107.10)	66.70 (45.20–109.20)	72.50 (45.20–588.70)*	<0.001
**IVSd (mm)**	8.39±1.06	8.59±0.83	9.22±1.11*^#^	<0.001
**LVIDd (mm)**	8.46±1.02	8.80±0.79*	9.45±1.17*^#^	<0.001

Values are given as mean ± SD, percentages or median [range].

Abbreviations: TOD, target organ damage; FH, familial history of hypertension; BMI, body mass index; SBP, systolic blood pressure; DBP, diastolic blood pressure; MAP, mean arterial blood pressure; CMV, cytomegalovirus; CRP, C-reactive protein; TNF-α, tumor necrosis factor-α; IL-6, interleukin-6; FBS, fasting blood sugar; TC, total cholesterol; TG, triglyceride; LDL-C, low-density lipoprotein cholesterol; HDL-C, high-density lipoprotein cholesterol; BUN, serum blood urea nitrogen; Cr, serum creatinine; IVSd, the inter-ventricular septum end-diastolic thickness; LVIDd, the left ventricular end-diastolic dimension.

*P<0.05 hypertension without TOD or hypertension with TOD vs. normotensive, respectively

^#^*P*<0.05, hypertension without TOD vs. hypertension without TOD.

The seroprevalence of CMV IgG was 97.16% (547/563), thereby possibly indicating a high prevalence of CMV infection in the Han Chinese population. No significant difference in CMV seropositivity status between the participant with and those without CMV infection were found (Table 3); however, but the CMV-specific IgG titers had significant differences.

**Table 3:** Clinical characteristics and laboratory values of study participants with and without serologic evidence of CMV infection.

Characteristics	CMV infection	No CMV infection	*P* value
**n**	547	16	
**Age (years)**	55.50±12.13	51.63±9.78	0.206
**Males (%)**	232 (42.41)	8 (50.00)	0.545
**Smokers (%)**	192 (35.10)	5 (31.25)	0.750
**Alcohol (%)**	135 (24.68)	4 (25.00)	0.977
**FH (%)**	134 (24.50)	6 (37.50)	0.236
**Weight (Kg)**	71.37±12.10	74.46±12.95	0.316
**Height (m)**	1.67±0.08	1.70±0.09	0.136
**BMI (kg/m**^**2**^**)**	25.64±3.15	25.75±2.91	0.890
**SBP (mmHg)**	150.56±26.21	147.13±24.34	0.605
**DBP (mmHg)**	89.63±15.10	85.44±13.92	0.273
**MAP (mmHg)**	109.90±17.86	106.13±16.96	0.404
**CRP (ug/ml)**	0.51 (0.36–0.90)	0.55 (0.20–1.23)	0.340
**TNF-α (ng/ml)**	0.17 (0.13–0.34)	0.18 (0.13–0.53)	0.484
**IL-6 (ng/ml)**	0.14 (0.11–0.22)	0.16 (0.05–0.49)	0.440
**FBS (mmol/L)**	5.26±0.88	4.97±0.59	0.185
**TC (mmol/L)**	4.29±0.98	4.31±0.90	0.934
**TG (mmol/L)**	1.50±0.79	1.76±0.78	0.197
**LDL-C (mmol/L)**	2.76±0.84	2.61±0.71	0.496
**HDL-C (mmol/L)**	1.13±0.32	1.15±0.28	0.746
**BUN (mmol/L)**	5.31 (3.32–7.52)	5.22 (1.98–91.40)	0.784
**Cr (umol/L)**	69.35 (48.30–88.20)	69.30 (43.10–588.70)	0.673
**IVSd (mm)**	8.84±1.12	9.06±1.18	0.426
**LVIDd (mm)**	9.01±1.16	9.19±1.11	0.550
**Brain infarction (%)**	193 (35.28)	2 (12.50)	0.059
**CAP (%)**	238 (43.51)	4 (25.00)	0.140

Values are given as mean ± SD, percentages or median [range].

Abbreviations: CMV, cytomegalovirus; FH, familial history of hypertension; BMI, body mass index; SBP, systolic blood pressure; DBP, diastolic blood pressure; MAP, mean arterial blood pressure; CRP, C-reactive protein; TNF-α, tumor necrosis factor-α; IL-6, interleukin-6; FBS, fasting blood sugar; TC, total cholesterol; TG, triglyceride; LDL-C, low-density lipoprotein cholesterol; HDL-C, high-density lipoprotein cholesterol; BUN, serum blood urea nitrogen; Cr, serum creatinine; IVSd, the inter-ventricular septum end-diastolic thickness; LVIDd, the left ventricular end-diastolic dimension.

**Fig 1:**
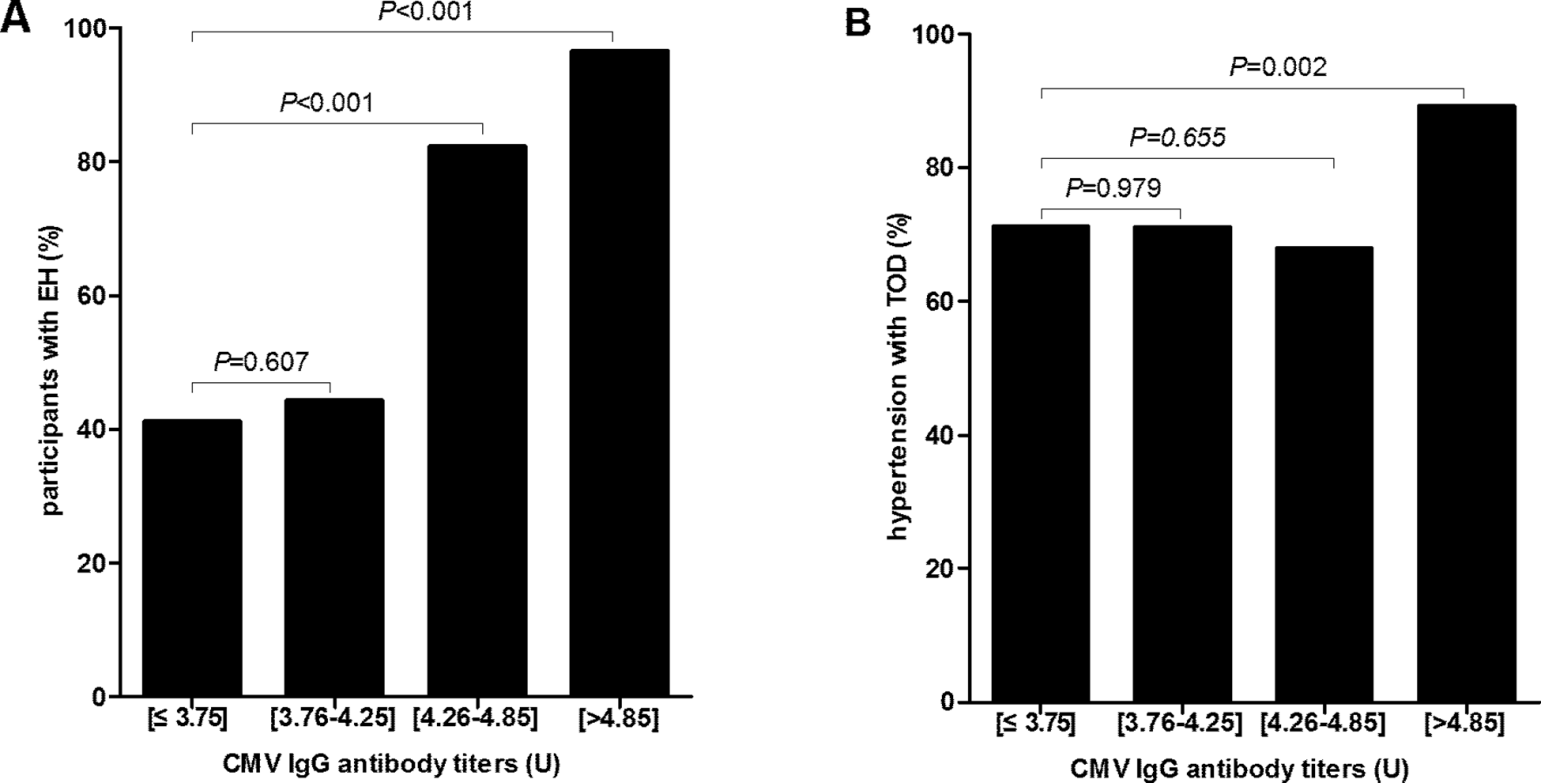
Incidence of hypertension and hypertensive TOD with different CMV IgG titers in study participants. Abbreviations: TOD, target organ damage; CMV, cytomegalovirus. A. Incidence of hypertension with different CMV IgG titers in all study participants. B. Incidence of hypertensive TOD with different CMV IgG titers in participants with hypertension.

In Fig 1, the subjects were divided into four groups, according to CMV antibody titers. CMV antibody titers were parameterized using a dummy variable to compare the highest quartile (quartile 4) with the bottom three quartiles (quartiles 1 to 3). Groups 1 to 4 had 143, 140, 136, and 144 subjects, and had 59, 62, 112, and 139 subjects with hypertension, respectively (Fig 1A and 1B). The prevalence of hypertension and hypertensive TOD were significantly different among the CMV IgG titer groups (*P*<0.001). Moreover, we examined the incidence of hypertension among the groups, and significant differences were observed between groups 1 and 3 as well as between groups 1 and 4 (see S1 Table for data) (*P*<0.001) (Fig 1A). The incidence of hypertension with TOD was significantly different between groups 1 and 4 (see S2 Table for data) (*P* = 0.002) (Fig 1B).

The correlation between CMV IgG titers and BP was confirmed by multivariable linear regression analyses (Table 4). CMV IgG titers were significantly positively correlated with BP (including SBP, DBP, and MAP) (*P*<0.001) in all models (model 1, before adjustment; model 2, adjustment for covariates: BMI, FBS, TG, HDL-C; model 3, further adjustment for age and sex). The CMV IgG titers had a significant positive correlation (*P*<0.001) with higher grade of BP and hypertension in all models, and were positively correlated with hypertension with TOD in models 1 and 2 (model 1, *P*<0.001; model 2, *P* = 0.013) (Table 5).

**Table 4:** Correlation of CMV IgG titers with BP in study participants.

	SBP		DBP		MAP	
	B	*P* value	B	*P* value	B	*P* value
**Model 1**	8.73	<0.001*	4.42	<0.001*	5.82	<0.001*
**Model 2**	7.08	<0.001*	3.43	<0.001*	4.61	<0.001*
**Model 3**	5.39	<0.001*	3.14	<0.001*	3.85	<0.001*

Abbreviations: CMV, cytomegalovirus; BP, blood pressure; SBP, systolic blood pressure; DBP, diastolic blood pressure; MAP, mean arterial blood pressure. Model 1: unadjusted for all the confounder factors; Model 2: further adjusted for BMI, FBS, TG, HDL-C; Model 3: further adjusted for age and sex.

*P<0.05 considered statistically significant.

**Table 5:** Correlation between CMV IgG titers and the different stages of BP and hypertension and hypertension without/with TOD in study participants.

	Model 1		Model 2		Model 3	
	r	*P* value	r	*P* value	r	*P* value
Group 1	0.59	<0.001*	0.45	<0.001*	0.37	<0.001*
Group 2	0.51	<0.001*	0.40	<0.001*	0.36	<0.001*
Group 3	0.20	<0.001*	0.18	0.001*	0.10	0.053

Abbreviations: CMV, cytomegalovirus; BP, blood pressure; TOD, target organ damage.

Group 1, the four grades of BP (from normotensive to grade 3 hypertension in sequence); Group 2, the three grades of the hypertension (from grade 1 hypertension to grade 3 hypertension in sequence); Group 3, the two grades of hypertension without/with TOD. Model 1: unadjusted for all the confounder factors; Model 2: further adjusted for BMI, FBS, TG, HDL-C; Model 3: further adjusted for age and sex.

*P<0.05 considered statistically significant.

To determine whether CMV IgG titer is an independent risk factor for the development of hypertension, we used binary logistic regression to adjust for potential confounding factors. We found that CMV IgG titers are associated with hypertension in all models (*P*<0.001). Compared with the lowest CMV IgG quartile (≤3.75 U), CMV IgG titers of quartiles 3 and 4 (4.26–4.85 and >4.85 U, respectively) were significantly associated with hypertension in all models (*P*<0.001). We further categorized the IgG titers into two groups (i.e., ≤4.25 and >4.25 U). CMV IgG titer >4.25 U was significantly associated with hypertension in all models (*P*<0.001) compared with CMV IgG ≤4.25 U (Table 6).

**Table 6:** Hazard ratio for incidence of hypertension by CMV IgG titers.

	Model 1		Model 2		Model 3	
	OR (95% CI)	*P* value	OR (95% CI)	*P* value	OR (95% CI)	*P* value
**CMV IgG titers (U)**	2.11 (1.75–2.54)	<0.001*	1.95 (1.59–2.40)	<0.001*	1.66 (1.33–2.06)	<0.001*
**The titers quartiles (U)**						
**≤3.75**	1 (reference)		1 (reference)		1 (reference)	
**3.76–4.25**	1.13(0.71–1.81)	0.607	0.97(0.55–1.70)	0.907	0.78(0.42–1.45)	0.432
**4.26–4.85**	6.64 (3.82–11.54)	<0.001*	6.83(3.60–12.93)	<0.001*	6.97(3.48–13.95)	<0.001*
**>4.85**	39.58 (15.27–102.57)	<0.001*	25.73(9.35–70.81)	<0.001*	12.89(4.48–37.08)	<0.001*
**The titers binary division (U)**						
**≤4.25**	1 (reference)		1 (reference)		1 (reference)	
**>4.25**	11.59 (7.38–18.19)	<0.001*	10.52 (6.29–17.60)	<0.001*	9.37 (5.35–16.44)	<0.001*

Abbreviations: CMV, cytomegalovirus; OR, odds ratio; CI, confidence interval.

Model 1: unadjusted for all the confounder factors; Model 2: further adjusted for BMI, FBS, TG, HDL-C; Model 3: further adjusted for age and sex.

*P<0.05 considered statistically significant

Table 7 shows the results of the ordinal logistic regression for the progression of hypertension. The CMV IgG antibody titer was a significant factor for the progression of hypertension in all models (*P*<0.001), and higher CMV IgG quartiles were significantly associated with higher grade of hypertension in all models (*P*<0.001). CMV IgG >4.25 U was significantly associated with higher grade of hypertension in all models (*P*<0.001) compared with CMV IgG ≤4.25 U.

**Table 7:** Hazard ratio for incidence of different grades hypertension by CMV IgG titers.

	Model 1		Model 2		Model 3	
	OR (95% CI)	*P* value	OR (95% CI)	*P* value	OR (95% CI)	*P* value
**CMV IgG titers (U)**	0.53(0.44–0.62)	<0.001*	0.54(0.45–0.65)	<0.001*	0.57 (0.47–0.68)	<0.001*
**The titers quartiles (reverse order) (U)**	2.17(1.78–2.63)	<0.001*	2.11(1.73–2.59)	<0.001*	2.02(1.64–2.48)	<0.001*
**The titers binary division (U)**						
**>4.25**	1 (reference)		1 (reference)		1 (reference)	
**≤4.25**	4.41 (2.89–6.76)	<0.001*	4.52 (2.90–7.04)	<0.001*	4.19 (2.67–6.57)	<0.001*

Abbreviations: CMV, cytomegalovirus; OR, odds ratio; CI, confidence interval.

Model 1: unadjusted for all the confounder factors; Model 2: further adjusted for BMI, FBS, TG, HDL-C; Model 3: further adjusted for age and sex.

*P<0.05 considered statistically significant.

Furthermore, CMV IgG titers were also associated with hypertensive TOD in all models (model 3, *P* = 0.046; others, *P*<0.001; Table 8). Compared with the lowest CMV IgG quartile (≤3.75 U), the CMV IgG titer of quartile 4 (>4.85 U) was significantly associated with hypertensive TOD in models 1 and 2 (model 1, *P* = 0.002; model 2, *P* = 0.007) but had no significant association (*P* = 0.155) with hypertensive TOD in model 3. CMV IgG >4.85 U was significantly associated with hypertensive TOD in all models (model 3, *P* = 0.027; others, *P*<0.001) compared with CMV IgG ≤4.85 U.

**Table 8:** Hazard ratio for incidence of hypertension with TOD by CMV IgG titers.

	Model 1		Model 2		Model 3	
	OR (95% CI)	*P* value	OR (95% CI)	*P* value	OR (95% CI)	*P* value
**CMV IgG titers (U)**	1.41 (1.17–1.69)	<0.001*	1.39(1.15–1.68)	0.001*	1.24 (1.00–1.52)	0.046*
**The titers quartiles (U)**						
**≤3.75**	1 (reference)		1 (reference)		1 (reference)	
**3.76–4.25**	0.99 (0.45–2.17)	0.979	0.961(0.43–2.13)	0.921	0.84(0.35–1.99)	0.689
**4.26–4.85**	0.85 (0.43–1.70)	0.655	0.87(0.43–1.75)	0.697	0.83(0.39–1.75)	0.615
**>4.85**	3.35 (1.54–7.28)	0.002*	2.99(1.36–6.58)	0.007*	1.86(0.79–4.38)	0.155
**The titers two division (U)**						
**≤4.85**	1 (reference)		1 (reference)		1 (reference)	
**>4.85**	3.62 (1.98–6.63)	<0.001*	3.23 (1.74–5.97)	<0.001*	2.14 (1.09–4.19)	0.027*

Abbreviations: CMV, cytomegalovirus; TOD, target organ damage; OR, odds ratio; CI, confidence interval.

Model 1: unadjusted for all the confounder factors; Model 2: further adjusted for BMI, FBS, TG, HDL-C; Model 3: further adjusted for age and sex.

*P<0.05 considered statistically significant.

Table 9 shows the correlation between CMV IgG titers and the levels of inflammatory cytokines (CRP, TNF-α, IL-6). The CMV IgG titers were positively correlated with CRP and IL-6 levels in all models (model 3 of IL-6, *P* = 0.001; others, *P*<0.001), whereas had no significant correlation with TNF-**α** levels was noted.

**Table 9:** Correlation of CMV IgG titers with the levels of inflammatory cytokines.

	CRP		TNF-α		IL-6	
	B	*P* value	B	*P* value	B	*P* value
**Model 1**	0.03	<0.001*	0.00	0.222	0.01	<0.001*
**Model 2**	0.02	<0.001*	0.00	0.214	0.01	0.001*
**Model 3**	0.02	<0.001*	0.00	0.681	0.01	<0.001*

Abbreviations: CMV, cytomegalovirus; CRP, C-reactive protein; TNF-α, tumor necrosis factor-α; IL-6, interleukin-6.

Model 1: unadjusted for all the confounder factors; Model 2: further adjusted for BMI, FBS, TG, HDL-C; Model 3: further adjusted for age and sex.

*P<0.05 considered statistically significant.

Table 10 shows the correlation between the levels of inflammatory cytokines (CRP, TNF-α, IL-6) and BP values. CRP levels were positively correlated with SBP in all models (model 1, *P*<0.001; model 2, *P* = 0.005; model 3, *P* = 0.038), DBP only in model 1 (*P* = 0.001), and MAP in models 1 and 2 (model 1, *P*<0.001; model 2, *P* = 0.024). TNF-α levels were positively correlated with SBP (all models, *P*<0.001), with MAP (model 1, *P* = 0.003; model 2, *P* = 0.001; model 3, *P* = 0.008), and with DBP only in models 1 (*P* = 0.049) and 2 (*P* = 0.030). IL-6 levels were positively correlated with SBP (model 1, *P*<0.001; model 2, *P* = 0.001; model 3, *P* = 0.018) and with MAP only in model 1 (*P* = 0.002). As the inflammatory cytokines levels were positively correlated with BP, we examined whether inflammatory cytokines levels were also positively correlated with higher grades of BP and hypertension and hypertension with TOD (Table 11). Moreover, CRP levels had a significant positive relationship with higher grade of BP and hypertension in all models (model 3 of group 1, *P* = 0.003; others, *P*<0.001) and with hypertensive TOD only in models 1 and 2 (model 1, *P* = 0.048; model 2, *P* = 0.0135). TNF-α levels had a significant positive relationship only with higher grade of BP in all models (model 3, *P* = 0.002; others *P*<0.001). IL-6 levels also had a significant positive relationship with higher grade of BP in all models (model 3, *P* = 0.022; others, *P* = 0.002) and higher grade of hypertension in model 1 (*P* = 0.024).

**Table 10:** Correlation of the levels of inflammatory cytokines with BP.

	SBP		DBP		MAP	
	B	*P* value	B	*P* value	B	*P* value
**CRP**						
**Model 1**	24.92	<0.001*	10.57	0.001*	15.24	<0.001*
**Model 2**	14.48	0.005*	4.54	0.125	7.76	0.024*
**Model 3**	9.66	0.038*	3.19	0.273	5.27	0.107
**TNF-α**						
**Model 1**	85.24	<0.001*	25.06	0.049*	45.03	0.003*
**Model 2**	85.61	<0.001*	24.73	0.030*	44.95	0.001*
**Model 3**	63.61	<0.001*	18.49	0.099	33.55	0.008*
**IL-6**						
**Model 1**	72.95	<0.001*	18.31	0.070	36.3	0.002*
**Model 2**	50.02	0.001*	5.12	0.574	19.87	0.061
**Model 3**	33.92	0.018*	0.54	0.952	11.509	0.254

Abbreviations: BP, blood pressure; SBP, systolic blood pressure; DBP, diastolic blood pressure; MAP, mean arterial blood pressure; CRP, C-reactive protein; TNF-α, tumor necrosis factor-α; IL-6, interleukin-6.

Model 1: unadjusted for all the confounder factors; Model 2: further adjusted for BMI, FBS, TG, HDL-C; Model 3: further adjusted for age and sex.

*P<0.05 considered statistically significant.

**Table 11:** Correlation between the levels of inflammatory cytokines and the different stages of BP and hypertension and hypertension without/with TOD in study participants.

	Model 1		Model 2		Model 3	
	r	*P* value	r	*P* value	r	*P* value
**CRP**						
**Group 1**	0.17	<0.001*	0.15	<0.001*	0.12	0.003*
**Group 2**	0.18	<0.001*	0.20	<0.001*	0.18	<0.001*
**Group 3**	0.10	0.048*	0.13	0.013*	0.10	0.057
**TNF-α**						
**Group 1**	0.15	<0.001*	0.17	<0.001*	0.13	0.002*
**Group 2**	-0.05	0.347	-0.04	0.454	-0.04	0.397
**Group 3**	0.06	0.245	0.08	0.149	0.08	0.151
**IL-6**						
**Group 1**	0.13	0.002*	0.13	0.002*	0.10	0.022*
**Group 2**	0.12	0.024*	0.10	0.064	0.08	0.153
**Group 3**	0.07	0.203	0.09	0.080	0.05	0.304

Abbreviations: BP, blood pressure; TOD, target organ damage; CRP, C-reactive protein; TNF-α, tumor necrosis factor-α; IL-6, interleukin-6

Group 1, the four grades of blood pressure (from normotensive to grade 3 hypertension in sequence); Group 2, the three grades of the hypertension (from grade 1 hypertension to grade 3 hypertension in sequence); Group 3, the two grades of hypertension without/with target organ damage. Model 1: unadjusted for all the confounder factors; Model 2: further adjusted for BMI, FBS, TG, HDL-C; Model 3: further adjusted for age and sex.

*P<0.05 considered statistically significant.

Table 12 shows the association of the levels of inflammatory cytokines (CRP, TNF-α, IL-6) with hypertension. CRP levels were associated with hypertension in model 1 (*P =* 0.003). No significant relationships between CRP levels and hypertension in models 2 and 3 were detected. TNF-α and IL-6 levels were associated with hypertension in all models (TNF-α: *P<*0.001; IL-6: model 1, *P =* 0.002; model 2, *P =* 0.029; model 3, *P =* 0.044).

**Table 12:** Hazard ratio for incidence of hypertension by the levels of inflammatory cytokines.

	Model 1		Model 2		Model 3	
	OR(95%CI)	*P* value	OR(95%CI)	*P* value	OR(95%CI)	*P* value
**CRP (ug/ml)**	4.24 (1.66–10.87)	0.003*	2.15 (0.73–6.35)	0.168	1.47 (0.43–4.97)	0.54
**TNF-α (ng/ml)**	2.41×10^5^ (1.87×10^3^−3.11×10^7^)	<0.001*	1.65×10^6^ (6.74×10^3^−4.06×10^8^)	<0.001*	4.96×10^5^ (1.98×10^3^−1.24×10^8^)	<0.001*
**IL-6 (ng/ml)**	138.08 (6.25–3.05×10^3^)	0.002*	43.86 (1.47–1.31×10^3^)	0.029*	58.36 (1.12–3.03×10^3^)	0.044*

Abbreviations: OR, odds ratio; CI, confidence interval; CRP, C-reactive protein; TNF-α, tumor necrosis factor-α; IL-6, interleukin-6.

Model 1: unadjusted for all the confounder factors; Model 2: further adjusted for BMI, FBS, TG, HDL-C; Model 3: further adjusted for age and sex.

*P<0.05 considered statistically significant.

The results of the ordinal logistic regression for the progression of hypertension are shown in Table 13. CRP level was a significant factor of the progression of hypertension in all models (*P*<0.001); apparently, TNF-α and IL-6 levels are not factors for progression of hypertension.

**Table 13:** Hazard ratio for incidence of different grades hypertension by the levels of inflammatory cytokines.

	Model 1		Model 2		Model 3	
	OR(95%CI)	*P* value	OR(95%CI)	*P* value	OR(95%CI)	*P* value
**CRP (ug/ml)**	0.09(0.03–0.25)	<0.001*	0.13(0.04–0.36)	<0.001*	0.15(0.05–0.44)	<0.001*
**TNF-α (ng/ml)**	10.06(0.32–321.18)	0.191	3.58(0.10–122.85)	0.479	4.19(0.12–150.51)	0.433
**IL-6 (ng/ml)**	0.04(0.00–0.0.64)	0.023*	0.07(0.00–1.33)	0.077	0.11(0.01–2.13)	0.145

Abbreviations: OR, odds ratio; CI, confidence interval; CRP, C-reactive protein; TNF-α, tumor necrosis factor-α; IL-6, interleukin-6.

Model 1: unadjusted for all the confounder factors; Model 2: further adjusted for BMI, FBS, TG, HDL-C; Model 3: further adjusted for age and sex.

*P<0.05 considered statistically significant.

Table 14 shows the association of the levels of inflammatory cytokines (CRP, TNF-α, IL-6) with hypertensive TOD. CRP levels were associated with hypertensive TOD in all models (model 1, *P* = 0.006; model 2, *P* = 0.021; model 3, *P* = 0.043). TNF-α and IL-6 levels were not significantly associated with hypertensive TOD.

**Table 14:** Hazard ratio for incidence of hypertension with TOD by the levels of inflammatory cytokines.

	Model 1		Model 2		Model 3	
	OR(95%CI)	*P* value	OR(95%CI)	*P* value	OR(95%CI)	*P* value
**CRP (ug/ml)**	6.60(1.70–25.63)	0.006*	6.10(1.49–24.93)	0.012*	4.95(1.05–23.36)	0.043*
**TNF-α (ng/ml)**	17.69(0.15–2112.29)	0.239	33.81(0.29–3993.55)	0.148	48.43(0.33–7195.37)	0.128
**IL-6 (ng/ml)**	53.37(1.01–2810.32)	0.049*	35.22 (0.66–1893.50)	0.080	18.46 (0.23–1462.91)	0.191

Abbreviations: TOD, target organ damage; OR, odds ratio; CI, confidence interval; CRP, C-reactive protein; TNF-α, tumor necrosis factor-α; IL-6, interleukin-6.

Model 1: unadjusted for all the confounder factors; Model 2: further adjusted for BMI, FBS, TG, HDL-C; Model 3: further adjusted for age and sex.

*P<0.05 considered statistically significant.

## Discussion

To the best of our knowledge, our study is the first to comprehensively report on the association of CMV IgG titers with the progression of hypertension and hypertensive TOD in the Han Chinese population. The involvement of CMV in the pathogenesis of hypertension is widely accepted and CMV has been linked to hypertension. Previous research found that CMV seropositivity is associated with hypertension [30]. Our previous study, Jeong SJ, and Simanek AM found that CMV IgG titers are associated with BP [13, 15, 31] and CMV infection may be a predisposing factor for hypertension [32]. Moreover, some studies also suggested that CMV plays an essential role in coronary artery disease [18, 33], carotid atherosclerotic plaques [19, 34], stroke [35, 36], CKD [37], and increased arterial stiffness in the early stage of CKD [38].

Although most of the aforementioned studies demonstrated a positive relationship between CMV infection or high CMV IgG levels and hypertension [13, 15, 30, 32], little is known about the effects of CMV infection and high CMV IgG levels on the progression of hypertension and hypertensive TOD. Nevertheless, CMV antibody may indirectly represent the cumulative viral burden [17, 39, 40]. Recently, Jeong SJ [31] and Simanek AM [13] showed that CMV antibody levels are positively correlated with BP, which is similar to our results. Moreover, a study showed that CMV IgG titer >127 U/ml might be an independent risk factor for diabetic atherosclerosis in type 2 diabetes mellitus [3]. In our study, we found that higher CMV IgG antibody titer is associated with hypertension, progression of hypertension, and hypertensive TOD. CMV IgG titer >4.25 U might be an independent predictor of hypertension and the progression of hypertension, and CMV IgG titer >4.85 U might be an independent risk factor for hypertensive TOD. Thus, CMV titers remain the strongest predictor of the progression of hypertension and hypertensive TOD.

We confirmed that CMV IgG antibody titers and BP are positively associated. Although the conclusion was similar to that of Jeong SJ [31] and Simanek AM [13], was inconsistent with our previous study [15]. This discrepancy may due to the difference in CMV infection which could be attributed to the following factors: hygienic conditions, lifestyle behaviors, socioeconomic status, age ranges, close contact with young children, and sexual exposure to multiple partners [3, 41]. CMV IgG antibody remains in the human host throughout his/her lifetime [16]. Recurrent CMV infection, reactivation of latent CMV, or underlying immune status may result in higher CMV IgG antibody concentrations [3, 17, 42]. CMV IgM antibodies may also suggest current infection [43]. In this study, we found that seropositive CMV IgG was higher than seropositive CMV IgM, which was scarce in all participants. Moreover, qPCR was employed to evaluate CMV DNA in blood specimens; CMV DNA was only detectable in 0.88% of all participants. Although no difference in CMV infection between the hypertension and normotension groups was noted, the CMV IgG titers were significantly related with hypertension, progression of hypertension, and hypertensive TOD. Additionally, our study suggests that latent and longer CMV infection could affect hypertension, progression of hypertension, and hypertensive TOD. However, whether high anti-CMV antibody titers were induced by frequent viral reactivations, longer infection, or underlying immune status remains to be further investigated. These findings could affect the reliability of our findings on the relationship between anti-CMV antibody levels and the progression of hypertension or hypertensive TOD. Nevertheless, reducing CMV exposure and frequence of the viral reactivations may be necessary among infected individuals.

Numerous studies support an independent association between CMV infections and hypertension. CMV infection affects the renin-angiotensin system (RAS), which could be linked to hypertension [30, 44]. CMV infection was found to increase arterial pressure, induce renin expression in kidney cells and vascular endothelial cells (EC), and increase angiotensin-II in mouse serum and arterial tissues [25]. CMV activity may lead to an increased RAS activation, resulting in arterial constriction via the influence of angiotensin-II, thereby increasing BP values [13]. Moreover, CMV could infect all cell types, including smooth muscle cells (SMC), EC, and leucocytes [45, 46]. Hence, CMV infections likely contributes to hypertension by vascular cell damage, consequently increasing oxidative stress levels and inflammation [47, 48]. In a previous study, increased oxidative stress in spontaneously hypertensive rats resulted in decreased nitric oxide (NO) bioavailability and soluble guanylyl cyclase expression and activity [49]. CMV infection impairs endothelial NO synthase function and causes endothelial damage [50], enhances NAD(P)H oxidase activity, and elicits reactive oxygen species (ROS) production. ROS directly causes vasoconstriction via the modulation of calcium levels and/or arachidonic acid metabolism [51, 52], and ROS contributes to collagen secretion by vascular smooth cells, which may favor the progression of intimal hyperplasia [53].

Moreover, the inflammatory cytokine CRP is considered to be an indicator of systemic low-grade inflammation [13] and an active mediator of cardiovascular disease [54]. Increased CRP levels are one of the strongest predictors of progressive vascular diseases [55]. In addition, CMV-infected cells also secrete a number of inflammatory cytokines, such as TNF-α and IL-6 [56]. CMV-independent TNF-a production may trigger latent CMV reactivation [57] and can affect the mechanical properties of arteries via increased stiffness of vascular endothelial cells and vascular permeability [58], thereby promoting vascular SMC proliferation [59]. IL-6 also promotes vascular SMC proliferation, which is a feature of the early stages of hypertension and atherosclerosis [60]. A combination of IL-6, high-sensitivity C-reactive protein, and high CMV-neutralizing antibody titers increases the risk of cardiac events [18]. A previous study also found that CMV infection increases BP apparently via the overexpression of inflammatory cytokines (including IL-6 and TNF-α) [25]. These mechanisms may partly explain the association we observed among CMV IgG titers, inflammation cytokine levels, higher grade of hypertension, and hypertensive TOD. Furthermore, we found that higher CMV IgG titer and CRP levels were an independent risk factor of hypertension progression and hypertensive TOD and that CMV IgG titers were positively correlated with CRP and IL-6 levels. These findings suggest that CMV infection likely contributes to the progression of hypertension and hypertensive TOD and is possibly mediated by inflammation.

This study has several limitations. First, the sample size was relatively small. Second, we only studied the Han Chinese population, which could not represent all Chinese populations or all ethnic groups. Third, our study is a cross-sectional study. Future studies should include a larger sample and a longer follow-up to strengthen the results. Nonetheless, this study has clinical significance. This is the first comprehensive report to evaluate the relationship between anti-CMV antibody titers and the progression of hypertension and hypertensive TOD in an Asian population.

In summary, our novel results demonstrate that CMV infection is associated with hypertension, progression of hypertension, and hypertensive TOD. CMV IgG titer >4.25 U could be an independent predictor of hypertension and even the progression of hypertension, while CMV IgG titer >4.85 U could be an independent risk factor for hypertensive TOD. The underling mechanism may be largely mediated by chronic inflammation. These findings provide insights into the role of CMV in the pathogenesis of hypertension progression and hypertensive TOD in the Han Chinese population.

## Supporting information

S1 Table Incidence of hypertension with different CMV IgG titers in study participants. (DOC)

S2 Table Incidence of hypertensive TOD among different CMV IgG titers in hypertension. (DOC)
