# Supplementary material for: High anti-human cytomegalovirus antibody levels are associated with the progression of essential hypertension and target organ damage in Han Chinese population
Source: PLoS One. 2017 Aug 24;12(8):e0181440. doi: 10.1371/journal.pone.0181440 (PMC5570371; doi:10.1371/journal.pone.0181440)
Supplement: S1 Table — (DOC) [file pone.0181440.s001.doc]

S1 Table. Incidence of hypertension with different CMV IgG titers in study participants.

| Characteritics | n | Participants with hypertension (%) |
| --- | --- | --- |
| Quartile 1 of CMV IgG titers | 143 | 59(41.26) |
| Quartile 2 of CMV IgG titers | 140 | 62(44.29) |
| Quartile 3 of CMV IgG titers | 136 | 112(82.35)^*^ |
| Quartile 4 of CMV IgG titers | 144 | 139(96.53)^#^ |

Abbreviations: CMV, cytomegalovirus.

Quartiles 1, 2, 3, and 4 of CMV antibody titers (U): 0-3.75, 3.76-4.25, 4.26-4.85, and >4.85, respectively.

^*^*P*<0.05 Quartile 3 vs. Quartile 1 of CMV IgG titers; ^#^*P*<0.05 Quartile 4 vs. Quartile 1.
