# Supplementary material for: High anti-human cytomegalovirus antibody levels are associated with the progression of essential hypertension and target organ damage in Han Chinese population
Source: PLoS One. 2017 Aug 24;12(8):e0181440. doi: 10.1371/journal.pone.0181440 (PMC5570371; doi:10.1371/journal.pone.0181440)
Supplement: S2 Table — (DOC) [file pone.0181440.s002.doc]

S2 Table. Incidence of hypertensive TOD among different CMV IgG titers in hypertension patients.

| Characteritics | n | Hypertension with TOD (%) |
| --- | --- | --- |
| Quartile 1 of CMV IgG titers | 59 | 42(71.19) |
| Quartile 2 of CMV IgG titers | 62 | 44(70.97) |
| Quartile 3 of CMV IgG titers | 112 | 76(67.86) |
| Quartile 4 of CMV IgG titers | 139 | 124(89.21) ^*^ |

Abbreviations: TOD, target organ damage; CMV, cytomegalovirus.

Quartiles 1, 2, 3, and 4 of CMV antibody titers (U): 0-3.75, 3.76-4.25, 4.26-4.85, and >4.85, respectively.

^*^*P*<0.05 Quartile 4 vs. Quartile 1 of CMV IgG titers.
